# Collagen Hydrolysates from Animal By-Products in Topical Cosmetic Formulations

**DOI:** 10.3390/ijms26062776

**Published:** 2025-03-19

**Authors:** Pavlína Egner, Jana Pavlačková, Jana Sedlaříková, Lucie Matošková, Pavel Mokrejš, Magda Janalíková

**Affiliations:** 1 Department of Fat, Surfactant and Cosmetics Technology, Faculty of Technology, Tomas Bata University in Zlín, Vavrečkova 5669, 760 01 Zlín, Czech Republic; sedlarikova@utb.cz (J.S.); l_matoskova@utb.cz (L.M.); 2 Department of Polymer Engineering, Faculty of Technology, Tomas Bata University in Zlín, Vavrečkova 5669, 760 01 Zlín, Czech Republic; mokrejs@utb.cz; 3 Department of Environmental Protection Engineering, Faculty of Technology, Tomas Bata University in Zlín, Vavrečkova 5669, 760 01 Zlín, Czech Republic; mjanalikova@utb.cz

**Keywords:** animal by-products, collagen hydrolysate, wrinkles, topical formulation, antimicrobial effect, bioengineering methods

## Abstract

The circular economy of animal by-products rich in collagen focuses on converting collagen into peptides with a defined molecular weight. Collagen hydrolysates prepared by biotechnological methods from chicken gizzards, deer tendons, and *Cyprinus carpio* skeletons can be an alternative source of collagen for cosmetic products that traditionally use bovine or porcine collagen hydrolysates. Collagen hydrolysates were characterized by antioxidant activity, surface tension, solution contact angle, and other parameters (dry weight, ash content, and solution clarity). Furthermore, the vibrational characterization of functional groups and their molecular weight was performed using the GPC-RID method. Subsequently, emulsion and gel cosmetic matrices were prepared with 0.5% and 1.5% collagen hydrolysates. Microbiological stability, organoleptic properties, and viscosity were investigated. Verification of the biophysical parameters of the topical formulations was performed in vivo on a group of volunteers by measuring skin hydration and pH and determining trans-epidermal water loss. Fish collagen hydrolysate was the most suitable for cosmetic applications in the parameters investigated. Moreover, it also effectively reduces wrinkles in the periorbital region when used in a gel matrix.

## 1. Introduction

As a biopolymer, collagen is a sustainable, available, biodegradable material. It possesses unique functional properties and exceptional biocompatibility, thus rendering it a raw material for versatile use in various products. The suitability of collagen as a biomaterial is primarily because it is a natural component of the extracellular matrix [1]. The ever-increasing global demand for collagen in the dental surgery, tissue engineering, bone grafting, pharmaceuticals, food, and cosmetic industries has been estimated at USD 6.63 billion by 2025, while the global market for marine collagen, for example, is expected to have an annual growth rate of 7.39% by 2026 [2]. The source of collagen can be categorized into natural sources of animal or plant origin or through recombinant protein production systems, including yeasts, bacteria, mammalian cells, insects, plants, and even artificial fibrils that mimic the properties of collagen [3]. The predominant source of collagen is from animal sources, such as cattle, pigs, chickens, marine, or freshwater organisms, and by-products generated during processing, including skin, hair, feathers, tendons, shells, fish scales, and bones [4,5]. Waste materials are minimized by recovering them to create high-value-added products [6,7]. Unfortunately, despite their availability and low cost, some sources can cause allergies or diseases such as osteogenesis imperfecta [3]. Without the risk of zoonoses such as spongiform encephalopathy (BSE) or foot-and-mouth disease (FMD), marine collagen appears to be safer [8]. Among non-animal collagens, there are studies dedicated to the production of recombinant collagens by bacteria such as *Streptococcus pyogenes*, *Methylobacterium* sp., *Solibacter usitatus*, and *Rhodopseudomonas palustris* [8,9], which could be a potential alternative to animal collagens for cosmetic applications. However, recombinant technologies are associated with high costs, low yields, and cofactors crucial for the stable formation of biofunctional and bioactive collagens. This fact again argues in favor of the application of animal collagen in research and clinical settings [3].

Collagen is the primary structural protein of the vertebrate body’s extracellular matrix, occurring in various morphologies in different tissues and thus fulfilling multiple biological functions [10]. The method of its preparation, rather than its source, is the factor that influences its physicomechanical properties. The helical structure is unique in the world of animal proteins. Each collagen molecule consists of at least 1000 amino acids and has an alpha-helical structure, contributing to its remarkable tensile strength, low extensibility, and high stability [11]. At the molecular level, collagen is characterized by covalent bonds that bind the atoms within the chains, while weaker intermolecular forces (hydrogen bonds, dipole–dipole forces, ionic bonds, van der Waals forces) arranged in a triple helical contribute to the overall structure of the molecule. The primary structure consists of a repeating sequence of identical amino acids, including glycine, proline, and hydroxyproline. However, lysine, hydroxylysine, alanine, aspartic acid, glutamic acid, and arginine may also be present [8]. Collagen is a highly cross-linked material with a high molecular weight of around 300–400 kDa [12], making it insoluble in water and oils. This property is problematic for the efficacy of cosmetics and food supplements. To overcome this issue, collagen is often modified into other forms [13]. During the thermal denaturation of collagen, weak bonds are broken, but covalent bonds remain intact. The temperature of thermal denaturation of collagen depends on the water content, the pH of the environmental medium, and the degree of cross-linking. When heated, the triple helix of the collagen unwinds, and the chains separate [14]. The denatured mass absorbs the water present and is called gelatine, whose molecular weight ranges from 15 kDa to 250 kDa [15]. For cosmetic applications, collagen is typically subjected to chemical or enzymatic hydrolysis to generate smaller peptides (usually up to 10 kDa) [16]. The peptides that are the shortest (below 500 Da), unlike native collagen, are well absorbed because they penetrate the skin easily. Cosmetic products can penetrate the skin directly across the *stratum corneum* by two pathways: the intercellular route, which involves diffusion between the lipid bilayers of the *stratum corneum*; and the transcellular route, which can be facilitated by liposomes, hydration, chemical enhancers, or physical methods such as microneedles and iontophoresis [16]. Water-soluble hydrophilic peptides are much easier to incorporate into formulation matrices. The hydrolyzed form also has a very low viscosity, and hydrolysis shifts the isoelectric point (pI) from values around pI = 7.0–8.0 to lower values around pI = 3.7–5.7 [14]. These changes depend precisely on the amino acid sequence according to the type and conditions of the hydrolysis process [17]. In addition, hydrolyzed collagen has antioxidant, immunomodulatory, antimicrobial, and anti-ageing properties [12].

Collagen, a key protein in skin firmness and elasticity, makes up 90% of the *dermis* [4,8]. Nevertheless, its production declines significantly with age—up to 1% per year after the age of 40. The ageing process is accompanied by the loss of collagen fibers, elastin, and hyaluronic acid, which leads to the formation of wrinkles [14]. Research shows that hydrolyzed collagen (in the molecular weight range of 1 kDa to 10 kDa) is a promising solution for slowing down this process due to its bioactivity and ability to stimulate fibroblasts to produce collagen and elastin. This collagen hydrolysate promotes skin regeneration, improves elasticity, and can positively affect nails and hair [14,16,17,18].

Based on the above facts, the efforts of the cosmetics industry are aimed at incorporating collagen biomolecules into the skin structure through cosmetic products or cosmeceuticals. The collagen supramolecule is used in these products as an active and functional ingredient, mainly to modify the textural properties. The hydrolyzed peptide is traditionally added to cosmetic formulations to moisturize and regenerate the skin. Collagen has an excellent water-binding capacity, and peptide occlusion reduces transepidermal water loss (TEWL) and forms a film that protects the skin and hair from potential mechanical damage or irritation caused by surfactants [3,19]. The highly competitive cosmetic market trend is to develop multifunctional products with added value. Therefore, collagen supramolecule is gradually being used in the attractive field of topical anti-aging products to mitigate the manifestations of extrinsic aging, mainly due to ultraviolet radiation [8]. For cost reasons, gelatine, a functional form of collagen, is often used as a viscosity regulator/thickener in cosmetic products [8]. Developing cosmetic products with collagen, as part of a strategy to control skin aging, focuses on optimizing its effect on the skin. Although collagen is already widely used, current studies on its effects are limited as patents protect it, and cosmetic companies use it to develop innovative formulations [20].

This study aimed to hydrolyze collagen from various animal sources enzymatically and to characterize the obtained hydrolysates. Cosmetic matrices incorporating these hydrolysates, such as emulsions and gels, were formulated for topical application. The efficacy of these formulations was evaluated in vivo, and the effect of collagen molecules in topical carriers was quantified using biophysical parameters of the skin.

## 2. Results

### 2.1. Dry Matter and Ash Content

The dry matter and ash content values are shown in Table 1. The determined dry matter was higher than 90% for all three CH samples, with the highest value for the fish hydrolysate.

The ash content indicates the content of inorganic substances extracted from feedstock. The lowest ash content of 0.66 ± 0.06% was found in CH from deer tendon, which refers to very pure material. On the other hand, the highest ash content of 24.89 ± 0.09% was found in CH from fish waste.

### 2.2. Clarity and pH

The clarity values of the CH solutions are noted in Table 2. The highest transmittance value was measured for deer CH (5.3%). This sample transmitted the most light and had the highest clarity. Purification further increased its clarity by 1.5%. The fish CH showed the lowest transmittance (0.9%), which increased by 1% during purification.

The CH solutions (6.67%) were subjected to pH measurements (Table 2). The deer CH had the highest pH value. Otherwise, the lowest was found in the fish CH. All pH values were between 5.0 and 7.5, the natural range of slightly acidic to neutral pH for skin.

### 2.3. Antioxidant Activity

The antioxidant activity (AA) of CH samples is presented in Table 3. The results showed that the fish CH at a concentration of 2 mg∙mL^−1^ (85.24%) exhibited the highest AA, while the deer CH (26.12%/10 mg∙mL^−1^) had the lowest AA among all CH samples. Thus, decreasing AA with increasing CH concentration can be observed. For chicken and deer CH samples, the AA value dropped steeply to almost 26%, while the fish CH still had 72.02% AA at 10 mg∙mL^−1^, so the decrease was more gradual.

### 2.4. Gel Permeation Chromatography (GPC)

The average molecular weight Mw and the number average molecular weight Mn were chosen to describe the molecular weight characteristics of the collagen hydrolysates, see Table 4. The lowest average molecular weight was found for the fish hydrolysate at 7.5 kDa. The polydispersity index PDI [21] was calculated from the Mw/Mn ratio. The deer collagen hydrolysate showed the most compact biopolymer molecular structure with PDI = 5.9.

### 2.5. Vibrational Characterization of Functional Groups

**Figure 1:**
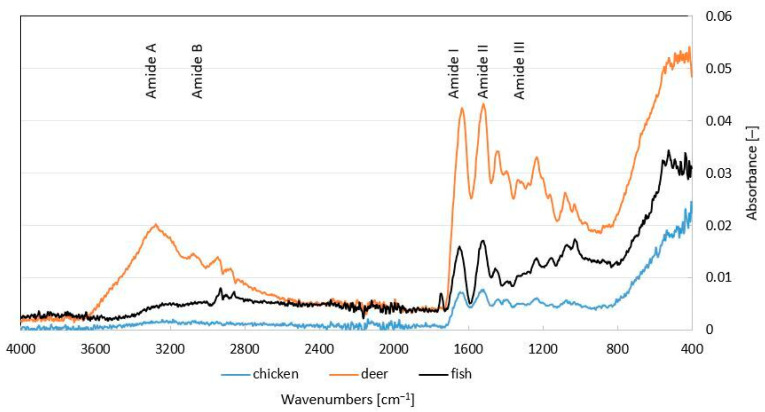
FTIR spectroscopy of collagen hydrolysate (chicken, deer, and fish).

The measured spectra are given in Figure 1. Typical bands for collagen gelatins or hydrolysates are denoted by amide A, amide B, and amide I–III with a specific range of wavenumbers in the IR spectra of the detected samples. The signal of amide A was observed at a peak of 3285 cm^−1^ corresponding to the N-H bond, the signal of amide B for the CH_2_ bond at 2864–2936 cm^−1^ for the deer collagen hydrolysate sample, and at 2852–2932 cm^−1^ for the fish hydrolysate sample. The presence of other peaks was also shown, i.e., in the amide I band for all characterized hydrolysates at 1603–1737 cm^−1^ for C=O bond, and in the amide II band at 1370–1570 cm^−1^ peak for -NH, COO-, CH_2_ bonds. In the lower signal region of the amide III band, a peak at 1333 cm^−1^ was observed in the deer hydrolysate sample. Other signals in the wave number range 1234–1238 cm^−1^.

### 2.6. Surface Tension and Contact Angle

**Figure 2:**
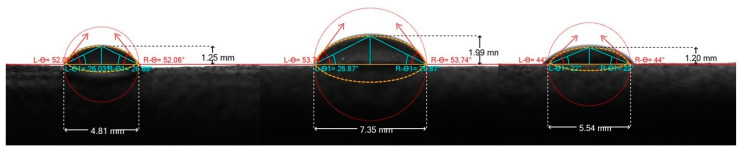
Contact angles of 1.5% CH solutions (from left chicken, deer, fish).

The measured surface tension values are reported in Table 5. The surface tension values for the CH samples ranged from 37.3 ± 0.5 mN∙m^−1^ (1.5% fish CH) to 48.8 ± 1.9 mN∙m^−1^ (0.5% chicken CH). In addition, the fish CH also showed quite low contact angle value (L-θ = 44.0°), indicating that this sample wets the surface very well. However, all CH samples in this study had contact angles below 90° indicating that they all wet skin surface very well (Figure 2).

### 2.7. Microbiological Analysis and Preservative Efficacy

The common microbial contamination was determined by the total viable counts of aerobic microorganisms and the total viable counts of yeasts and molds. No microbial growth was observed; thus, the contamination of each sample was lower than 10 CFU·g^−1^.

The preservative efficacy test with *Pseudomonas aeruginosa* ATCC27853 was conducted according to ISO 11930:2019, and the results of CH emulsions are summarized in Table 6. This method served to evaluate the antimicrobial protection of cosmetic products. Initial loads of *Pseudomonas aeruginosa* (approx. 5 LogCFU·g^−1^) in CH emulsions were gradually reduced. After 28 days, they were inhibited to less than 1 Log CFU·g^−1^, which is the detection limit of the method. However, only deer CH in both concentrations and 1.5% of fish CH met the criteria for microbiological safety criteria according to ISO 11930:2019 [22]. Other CH emulsions showed an acceptable level of protection, and the microbiological risk is considered tolerable for low-risk cosmetic products, such as those with an airless pump.

In contrast, all CH gel formulations immediately reduced the bacterial load to the detection limit, indicating inhibition of at least 5 log CFU·g^−1^ *P. aeruginosa*; thus, all CH gel formulations met the ISO criteria for microbiological safety.

The control samples (emulsions and gels) without the preservative but with collagen hydrolysates (chicken, deer, fish) were also tested with the same bacteria *Pseudomonas aeruginosa*. The results showed that even after 28 days of storage, *P. aeruginosa* grew very well at levels above 6 LogCFU·g^−1^. This means the preservative system is necessary, although its efficacy differs in the gels and emulsions.

### 2.8. In Vivo Efficacy of CH Emulsion and Gel Matrices on Volar Forearm Skin

**Figure 3:**
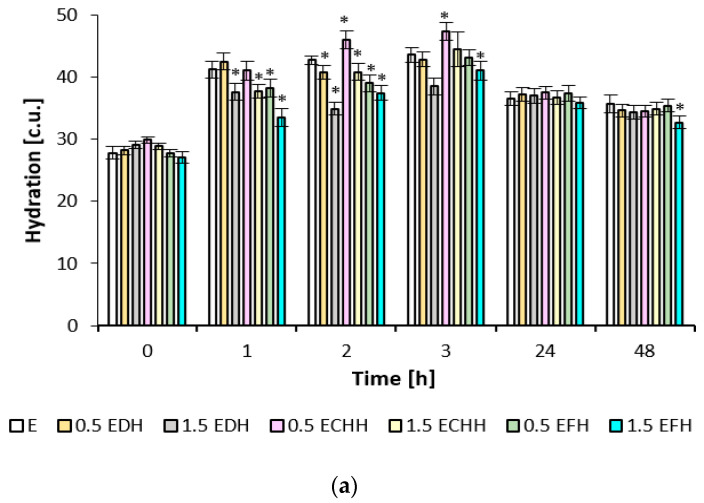

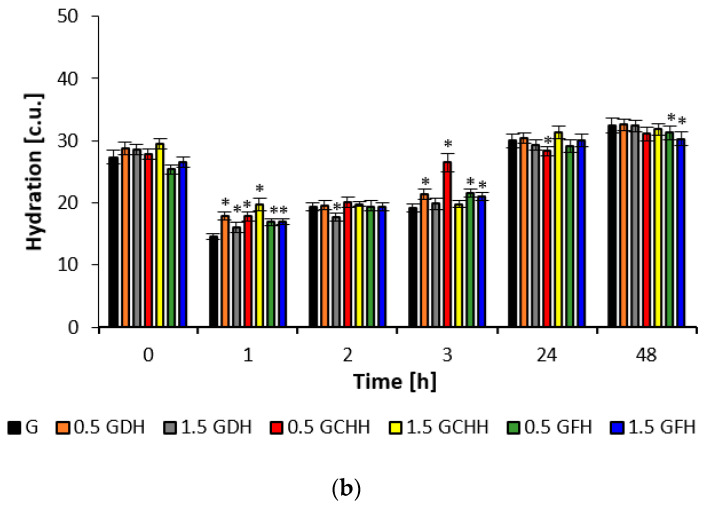
Hydration potential of (**a**) emulsions and (**b**) gels (E—emulsion without the addition of collagen hydrolysate; EDH—emulsion with the addition of deer collagen hydrolysate; ECHH—emulsion with the addition of chicken collagen hydrolysate; EFH—emulsion with the addition of fish collagen hydrolysate; G—gel without the addition of collagen hydrolysate; GDH—gel with the addition of deer collagen hydrolysate; GCHH—gel with the addition of chicken collagen hydrolysate; GFH—gel with the addition of fish collagen hydrolysate). * indicates statistically significant difference E or G vs. formulation with addition of collagen hydrolysate.

**Figure 4:**
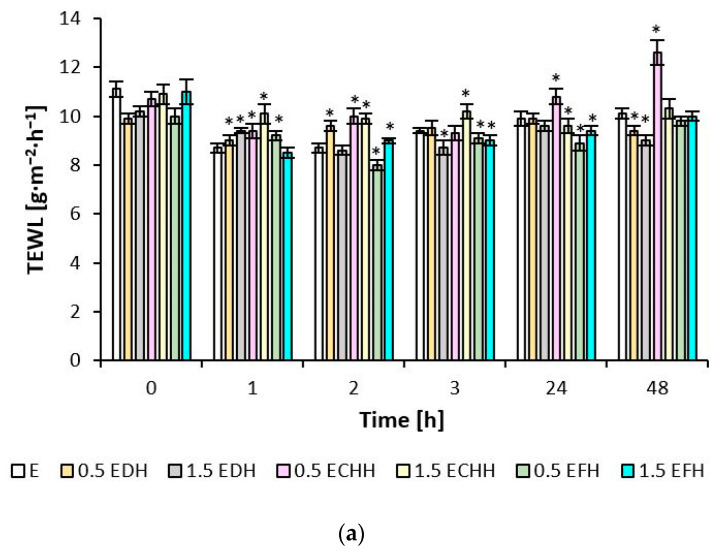

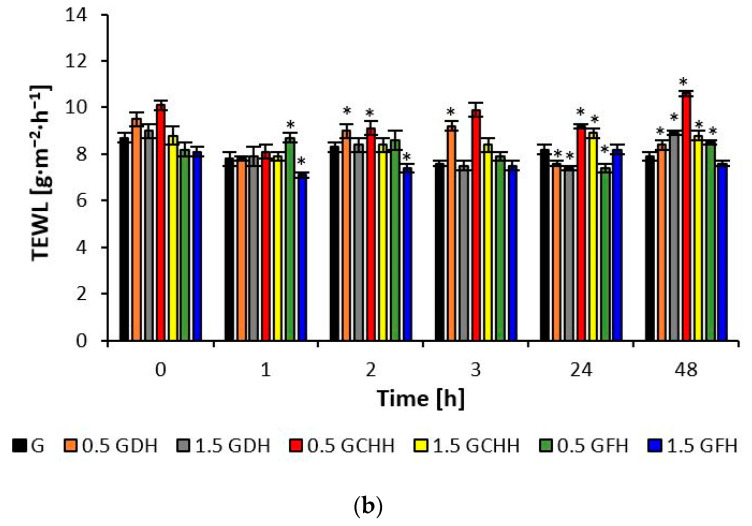
Barrier potential of (**a**) emulsions and (**b**) gels (E—emulsion without the addition of collagen hydrolysate; EDH—emulsion with the addition of deer collagen hydrolysate; ECHH—emulsion with the addition of chicken collagen hydrolysate; EFH—emulsion with the addition of fish collagen hydrolysate; G—gel without the addition of collagen hydrolysate; GDH—gel with the addition of deer collagen hydrolysate; GCHH—gel with the addition of chicken collagen hydrolysate; GFH—gel with the addition of fish collagen hydrolysate). * indicates statistically significant difference E or G vs. formulation with addition of collagen hydrolysate.

**Figure 5:**
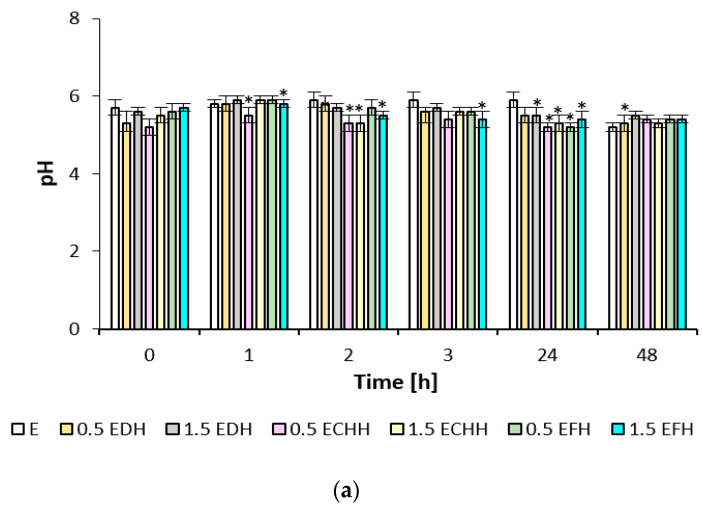

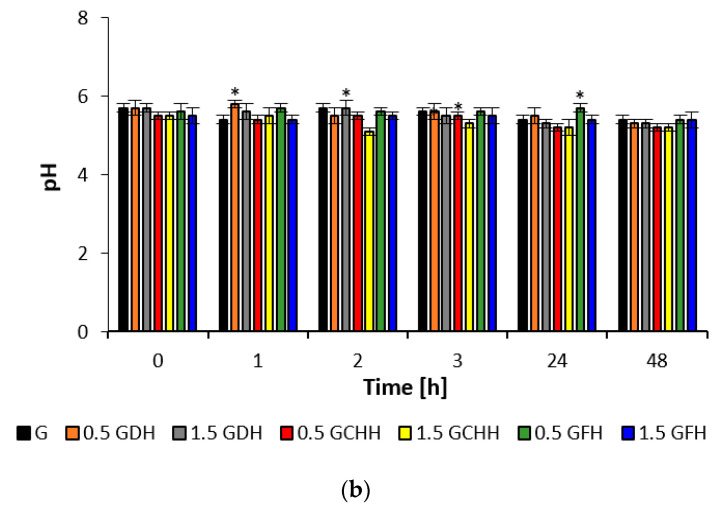
Skin pH values after application of (**a**) emulsions and (**b**) gels (E—emulsion without the addition of collagen hydrolysate; EDH—emulsion with the addition of deer collagen hydrolysate; ECHH—emulsion with the addition of chicken collagen hydrolysate; EFH—emulsion with the addition of fish collagen hydrolysate; G—gel without the addition of collagen hydrolysate; GDH—gel with the addition of deer collagen hydrolysate; GCHH—gel with the addition of chicken collagen hydrolysate; GFH—gel with the addition of fish collagen hydrolysate). * indicates statistically significant difference E or G vs. formulation with addition of collagen hydrolysate.

The effect of CH emulsion and gel matrices applied to the volar forearm skin was quantified using the biophysical parameters of hydration, TEWL, and pH, as shown in Figure 3, Figure 4 and Figure 5. The measured values indicate the different potential of the cream and gel formulations.

The improvement in skin hydration occurred after the application of emulsion formulations containing different types of collagen hydrolysates, with the asterisk indicating a statistically significant (*p* < 0.05) increase of up to 45% on average in the first three hours (Figure 3). In contrast to the effect of the gel formulations, skin hydration decreased during the first three hours, but an increase of up to 14% was observed 24 and 48 h after application. Thereafter, the observed hydration efficiency in the last two hours was only about 2–8 c.u. lower for the gel formulations than the emulsion formulations. The values for 0.5% fish collagen in the gel matrix and the 1.5% fish collagen concentration in both the emulsion and the gel matrix were statistically significant (*p* < 0.05) when tested against placebo in this time interval. After treatment with the emulsion matrices, the skin condition corresponded to a normally hydrated skin condition at all monitored values according to the instrumented scale; see the description in Section 4.7.3.

When the skin of the volar side of the forearm was treated with the prepared samples of both the emulsion and gel formulations, no TEWL values (Figure 4) were observed outside the range according to the scale given in Section 4.7.3, which would correspond to a disruption of the skin barrier, despite pretreatment with 0.5% SDS solution. At the observed time points, the values for epidermal water loss varied between 8.5 and 11.1 g·m^−2^·h^−1^ after application of the emulsions with hydrolysates and between 7.1 and 10.6 g·m^−2^·h^−1^ after application of the gels. The improvement of the barrier function fluctuated more frequently after applying matrices with deer and chicken CH compared to fish hydrolysate in the time intervals.

The effect of the prepared matrices on the skin’s pH value is documented in Figure 5. The initial acidity of the skin after treatment with SDS solution was between 5.2 and 5.7. The skin treated with emulsion matrices showed a pH increase of about 0.3 after the first hour; after treatment with gel formulations, the skin’s pH remained almost unchanged. In the subsequent time intervals, a decrease in skin pH can be observed, which can be attributed to applying all matrices with CH in the range of 0.1–0.6 up to the range of physiological values.

### 2.9. In Vivo Efficacy of Fish CH Gel Matrices on Skin of Periorbital Region

**Figure 6:**
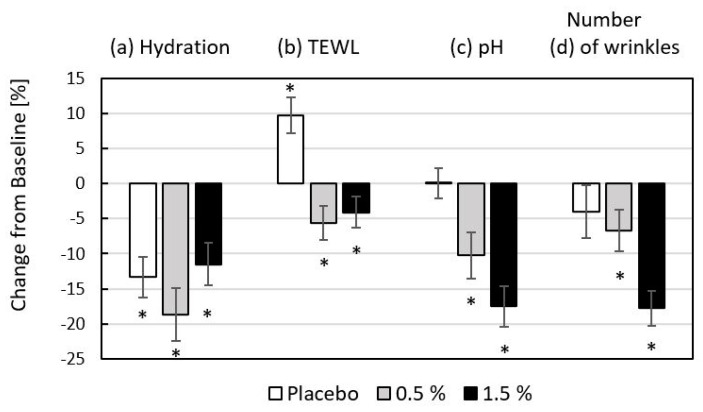
Percentage change of skin parameters: (a) hydration, (b) TEWL, (c) pH, and (d) number of wrinkles expressed as the arithmetic mean of the measured values of the right and the left periorbital parts of the face between baseline (T0) and the end of the eight weeks interventional period (T8) for placebo and 0.5% and 1.5% content of fish collagen hydrolysate in the gel matrix. * indicates significant changes of absolute values for *p* < 0.05.

The efficacy of the tested products is summarized in Figure 6, along with the differences between the changes (T8–T0) in skin hydration, TEWL, pH, and the number of wrinkles. In the group of volunteers using gel-placebo alone for periorbital care, there was a statistically significant (*p* < 0.05) decrease in skin hydration of 13.3%; the application of a gel containing 0.5% fish hydrolysate decreased skin hydration by 18.7%; and for the higher concentration of 1.5%, there was a decrease of 11.5%. TEWL was the next parameter monitored to assess skin barrier function, and statistically significant (*p* < 0.05) changes were also observed, with a decrease of 4.1–5.6% for skin treated with 0.5% and 1.5% hydrolysate formulations. Conversely, a 9.7% higher water loss was observed for the placebo. The acidity of the dermal mantle was within the range corresponding to physiological pH for the duration of this part of the experiment; the greatest change in skin pH was observed for the 1.5% fish hydrolysate formulation; and for both hydrolysate additions, the change in skin pH was significant (*p* < 0.05). Fish collagen hydrolysate proved highly effective in reducing the scanned number of wrinkles.

**Figure 7:**
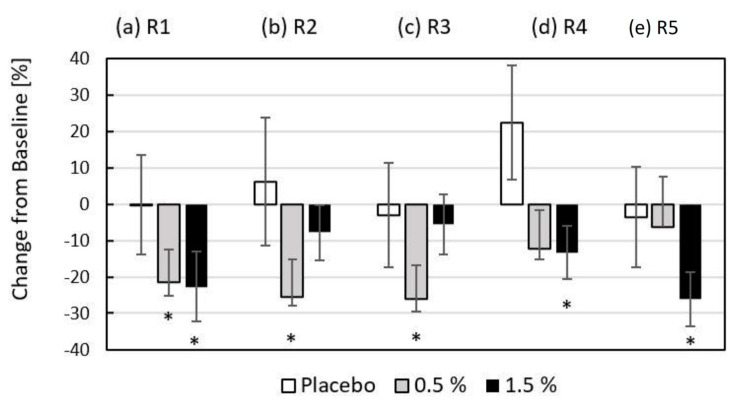
Percentage change of skin microrelief with values of roughness parameters: (a) R1, (b) R2, (c) R3, (d) R4, and (e) R5 expressed as the arithmetic mean of the measured values of the right and the left periorbital parts of the face between baseline (T0) and the end of the eight weeks interventional period (T8) for placebo and 0.5% and 1.5% content of fish collagen hydrolysate in the gel matrix. * indicates significant changes of absolute values for *p* < 0.05.

The results presented in Figure 7 show the quantitative change achieved by applying the gel matrix with/without hydrolysate to the skin of the periorbital facial region of the volunteers after eight weeks of use (T8–T0). The graph presents the average percentage change in the skin microrelief of the right and left periorbital region expressed by the parameters R1–R5, where R1 ≥ R2 ≥ R3 ≥ R4 ≥ R5. Significant alleviation of the change in skin roughness was observed with the use of formulations containing 0.5% (R1 by 21.4%, R2 by 25.5%, and R3 by 26.2%) and 1.5% (R1 by 22.7%, R4 by 13.3% and R5 by 26.1%) fish hydrolysate.

### 2.10. Organoleptic Properties

The results of the rank order tests are presented in Table 7 as rank sums for the parameters considered. Gel’s samples G, D, A, B, E, C, and F were evaluated as the best spreadable. At a significance level of *p* < 0.01 via the Neményi method, differences were found between samples AF, DF, and FG, with sample F always selected as the worst spreadable. For the emulsion formulations, the samples were in the order C, D, E, A, F, B, and G. Further statistically significant differences were found for this parameter between the pairs BC, CF and DG, with sample C being considered more spreadable than samples B and F and sample D more spreadable than sample G. Sample G was identified by the assessors as the most absorbable gel sample, followed by samples A, B, E, D, C, and F.

The existence of a difference between pairs of gels was determined between samples BF, AF, EF, and FG, of which sample F was identified as less absorbable than samples B, A, E, and G. Of the emulsion formulations, samples C and D were found to be the most absorbable, followed in order by samples A, E, F, B, and G. Significant differences were found between pairs BC, BD, AG, and DG, of which sample B is less spreadable than samples C and D and sample G is less spreadable than samples A and D.

The order of the gel samples evaluated for the odor was A, C, D, B, F, E, and G. A difference was found between the gel pairs AE, AF, AG, and CG. Sample A was rated as more pleasant in terms of odour than samples E, F, and G. Sample C was rated as more pleasant than sample G. For the emulsion formulations, the order of the samples was C, E, D, A, F, G, and B. Sample B was ranked as less pleasant compared to samples C, D, and E. Sample C was ranked as more pleasant smelling than sample F.

The evaluators ranked the gel samples in order of colour as A, B, C, E, D, F, and G. Neményi’s test revealed significant differences between samples AD, AE, AF, AG, BF, BG, and CG. Sample A was lighter in colour than samples D, E, F, and G. Sample B was lighter in colour than samples F and G; and sample C was lighter in colour than sample G. The emulsion formulations were in the order C, D, A, E, F, B, and G. A difference was found between pairs AG, BA, BD, and DG. Samples B and G were darker than samples A and D.

Regarding overall user preference, the evaluators ranked the gel formulations samples A, D, C, B, E, G, and F. A difference was found between samples AF, AG, and DF. Sample A was preferred by the users over samples F and G. For pair DF, sample D was preferred. Differences between the emulsion formulations ranked C, D, A, E, F, B, and G were shown by the pairs BC, BD, AG, DG, and EG. Sample B was less preferred than samples C and D, and sample G was less preferred than samples A, D, and E.

## 3. Discussion

The ash content value of fish skeletons CH was considerably high (24.89 ± 0.09). This could be mainly due to the origin of the raw material. It was a mixture of waste tissues, including bones. In a study by Kassem and El-Shemy [23], acid and alkaline extraction extracted protein hydrolysate from tanned hide waste. The amount of ash was 10.28%. The amount of ash in samples of hydrolysates from chicken gizzards in the study by Prokopova et al. [24] was determined to be 0.539 ± 0.007% under similar conditions (Protamex enzyme—0.15%; extraction temperature—62.5 °C). The difference in values may be due to the different physiological states of the animal, such as age.

The collagen peptides with the sequence of the repetitive amino acids (especially hydrophobic glycine and protein) show antioxidant activity. However, some aromatic amino acids and histidine also play an important role. Nonetheless, the exact mechanism of antioxidant action is unknown [16].

The results of the present study showed that fish CH had the best AA with increasing concentrations of CH in water, ranging from 72 to 85%. In the study by Xu et al. [25] the antioxidant activity of deer tendon collagen hydrolysate solutions was also investigated using the DPPH method in the concentration range of 0.5 to 7.5 mg∙mL^−1^. The highest AA was measured in samples treated with ultrasound for 60 min. As the CH concentration increased, the antioxidant activity increased, ranging from 20 (0.5 mg∙mL^−1^) to 70% (7.5 mg∙mL^−1^). In the study by Prokopova et al. [26], the antioxidant activity of collagen hydrolysates from chicken gizzards was investigated with DPPH and ABTS. Solutions of collagen hydrolysates were measured at concentrations of 2, 4, 6, 8, and 10 mg∙mL^−1^. AA values ranging from 70 to 77% were determined using the DPPH method. The increase in AA occurred with increasing concentration of collagen hydrolysate in the solution. Using the ABTS method, values between 82% and 95% were determined, whereby the increase was again dependent on the increase in the hydrolysate concentration in the solution. Yu et al. [27] extracted collagen products from fish skins (perch, grass carp, and tilapia) using five different methods: dry salt, wet salt, pepsin, acid, and temperature. The antioxidant activity of 10% hydrolysate solutions in demineralized water was determined using the DPPH and FRAP methods. The collagen products extracted with dry salt showed the highest antioxidant activity, with values above 75%.

The polydispersity index PDI was calculated from the Mw/Mn ratio to represent the degree of order of the system, with monodisperse systems having a PDI of 1. The index increases with the system’s increasing polydispersity [21]. The distribution of molecular weights of the collagen hydrolysate samples studied reflects the different sources used to produce the collagen hydrolysates. The influence of the hydrolysis itself on the splitting and cleavage of the collagen fibers was also evident. The influence of the raw material, the hydrolysis reagent, and technological production conditions is evident from the studies [25,28,29] showing the molecular weight fractions. The width of the molecular weight distribution and the resulting PDI can be decisive parameters for various applications, e.g., cosmetics, food, or pharmaceuticals, and for the different administration of bioactive substances [21].

Fourier transform infrared spectroscopy helps to elucidate changes in the secondary structure of collagen and gelatin. It is used to study collagen cross-linking, denaturation processes, thermal self-assembly, and gelatin melting. The FTIR spectra are correlated with amide groups that influence the secondary structure of the polypeptide and the amino acid arrangement within the collagen structure. The FTIR spectra showed characteristic peaks for collagen amides A, B, I–III, which indicate the properties of amino acids; in particular, amino acids such as proline and hydroxyproline found in collagen [30] exhibited bands corresponding to the five main amine groups at various wave numbers. There are differences between the samples. Amide A signals were observed only in deer hydrolysate, which is related to N–H stretching linked via hydrogen bonding, corresponding to the primary structure of collagen, indicating a higher presence of amino groups. The detected peaks of amide B in deer and fish hydrolysates are associated with the asymmetric elongation of -CH2. The amide I–III bands were detected in all hydrolysate samples analyzed by spectroscopy. Amid I corresponds to the stretching vibrations of C=O bonds associated with the bending vibrations of N−H and C−N stretching over C−N deformation. Amide II represents the bending vibration of N−H linked to C−N. Amid III displays a complex combination of α-helices, ß-sheets, and a random coil within the structure of a protein [29,31], indicating the triple helix structure in the extracted collagen [29,32]. The uncontrolled hydrolysis process unravels collagen fibers and breaks, which can vary depending on the type and age of the raw material. The samples, therefore, have different spectra and correspondingly different molecular weights, which is related to the applications of these low-molecular-weight products in cosmetics and their ability to penetrate the skin barrier or form an occlusive film, which positively decreased TEWL. In a study by Chanmangkang et al. [29], typical peaks (amides A, B, I, II, and III) were identified, almost identical to the peaks of the tested samples, demonstrating the presence of triple helix collagen fibers. In study [33], the IR spectra in the wavenumber range 450–4000 cm^−1^ were measured to determine the presence of bonds in collagen hydrolysates obtained by enzymatic extraction with papain and bromelain from domestic buffalo. The detected spectra of the analyzed samples correspond to those of the studies mentioned above, except for the peaks typical of collagen in the same wavenumber bands.

The common microbial contamination was not proved by determining total viable counts of aerobic microorganisms (TVC) and total viable counts of yeasts and molds (YMC). Therefore, it can be concluded that the microbial contamination of prepared cosmetic emulsion and gel formulations was lower than 10 CFU·g^−1^. However, it was described that collagen hydrolysate from chicken gizzards can be a source of bacteria, e.g., *Brevibacillus agri*, *Bacillus flexus*, *Acinetobacter radioresistens*, *Acinetobacter baumanii*, *Enterococcus faecium*, or *Staphylococcus hominis* [26]. On the other hand, the presence of pathogenic bacteria, such as *Salmonella*, *Escherichia coli*, *Pseudomonas aeruginosa*, *Bacillus cereus*, or *Listeria monocytogenes*, whose presence is prohibited in food and cosmetic applications by the EU Regulations [34,35], was not demonstrated. Similarly, the emulsion systems containing collagen hydrolysate were microbiologically tested for the presence of aerobic bacteria and yeasts [36]. Only very low counts (less than 2 LogCFU·g^−1^) of aerobic microorganisms were found, while the presence of yeasts and molds were not detected.

The preservation efficacy test was performed to evaluate the preservation of cosmetic emulsion and gel formulations. The whole inoculum *P. aeruginosa* was inhibited by CH gel formulations to less than 1 LogCFU·g^−1^. On the other hand, *P. aeruginosa* inoculum survived in CH emulsion formulations. However, all bacteria were gradually reduced to less than 1 LogCFU·g^−1^ during 28 days. Both emulsion and gel formulations contained the same concentrations of animal collagen hydrolysate, but their performance in the ISO 11930 test differed. While gels consistently achieved the required ≥3 Log reduction, emulsions (0.5% and 1.5% chicken CH samples and 0.5% fish CH sample) failed on day 14 but met the threshold on day 28. This could be due to interactions of the preservative with the oil phase in emulsions, temporarily reducing the antimicrobial efficacy. Similarly, a prolonged antimicrobial effect has been observed with 14% ethanol [37]. It is also more likely that the preservative is more unevenly distributed in emulsions than in gels. In addition, the gel’s viscosity may limit bacteria’s mobility, improving preservation. Although the delayed reduction in emulsions is expected, its compliance with the safety requirements of the final product must be carefully assessed.

The inner side of the volar forearm and the periorbital area were selected to test the effect of collagen hydrolysates on biophysical parameters and skin microrelief. In both areas, the efficacy of cosmetic matrices is most frequently tested on the skin. Regulation (EC) No 1223/2009 of the European Parliament and of the Council [35] classifies cosmetics, particularly for the eye area, as a cosmetic product to be specifically monitored. Cosmetic care in this area must be carried out in the knowledge that it is close to the boundary between the external covering of the body and its internal environment. In addition, the periorbital area is an important part of the face from a cosmetic point of view, as it shapes the perception of age and beauty. The skin around the eyes has fewer sweat and sebaceous glands and is thinner, with a higher density of blood and lymphatic vessels. Skin care in this area should consider age and gender, skin type, skin turgor, skin pigmentation, degree of dehydration, and the condition of the eyelids [38]. A key feature of this area is periorbital wrinkles, one of the first typical aging skin changes [39]. Periorbital rejuvenation aims to restore the appearance and minimize and soften wrinkles. It is recommended to use more nourishing products when growing older to treat fine lines and firm the skin and later to contour firmness and care for deep wrinkles [40]. Periorbital skin rejuvenation with topical products can help to improve the overall appearance. Compared to creams, gels represent a suitable non-greasy vehicle for accessible areas with easy application. Therefore, the gel was chosen for the test performed in the periorbital area [41]. This vehicle was preferred for its simple composition without any unnecessary ingredients. There is only one active ingredient, i.e., collagen hydrolysate, compared to the emulsion formulation. The use of gel formulations was based on the previous successful work of the authors [42] describing the effect of a gel matrix containing 1% chicken gizzard hydrolysate.

Although the emulsion formulation was more effective in terms of hydration parameters in in vivo tests on the volar forearm during a 48-h period, the gel matrix was chosen for the eye area, which is more recommended for the sensitive eye area and from a dermatological point of view than the emulsion matrix [43]. From a cosmetic science perspective, fish collagen was chosen for validation as it proved to be the most suitable for parameters such as lowest molecular weight, highest antioxidant activity, and TEWL. Another reason for choosing the gel base was the possibility of comparison with the results of a study conducted by our research team [42], which described the effect of a gel matrix containing 1% chicken gizzard hydrolysate.

Gel matrices containing a film-forming carbomer and the addition of collagen hydrolysate enhance the film-forming properties [44]. Film-forming agents probably caused a decrease in hydration of the volar forearm skin during the first three hours. Nevertheless, the gel film can positively affect skin moisture. A decrease was also observed in the periorbital region. However, despite the decrease in corneometric values (see Figure 3a and Figure 3b), it must be pointed out that the skin condition corresponded to a normal hydration level, i.e., above 45 c.u. [45]. In the aforementioned study by Prokopova [42], hydration values corresponding to normal skin hydration status were measured in the periorbital region of 10 volunteers, but with an increase of up to 12% after 8 weeks of application.

The TEWL parameter quantified the status of the skin barrier. The *stratum corneum* represents the core layer that regulates the passive diffusion of water from the deeper layers of the *epidermis*, which are very well-hydrated. The level of water flux through each structural layer decreases towards the skin surface. The film-forming properties of the gel matrix and collagen hydrolysate described in the study [20], together with a healthy *stratum corneum*, prevented the evaporation of epidermal water. The formation of a surface film on the inner side of the volar forearm and the periorbital region treated with formulations containing 0.5 and 1.5%, mainly fish CH, contributed to the reduction in monitored TEWL values. Placebo formulations did not reduce TEWL of the periorbital area. Still, they monitored values of this parameter were in the range of 15–25 g·m^−2^·h^−1^ for all formulations tested, indicating a healthy, normally functioning skin barrier. In study [46], presenting the results of hyaluronic acid, glycerol, and *Centella Asiatica* on the volar surface of the forearm, a reduction in TEWL values of 52% at 1 h, 32% at 8 h, and 48% at 24 h after application of the formulation is reported. Topical application of some natural ingredients can restore or improve the epidermal permeability barrier relatively quickly and thus reduce TEWL in intact skin, which proves to be very suitable for treating easily vulnerable areas [47].

The acidity of the skin barrier plays a very important role in the healthy functioning of the skin barrier and microbial protection. When measuring the changes after application of the prepared hydrolysate formulations in the skin of the volar side of the forearm, the values were in the range of pH 5.2–5.7 and in the periorbital area pH 4.5–5.5, corresponding to physiological values [48]. Thus, there was no adverse effect of the prepared formulations. A similar effect was observed in studies [49,50] using natural ingredients such as sea sponge extract, honey, beeswax, royal jelly, and propolis.

Lu et al. [51], who investigated the efficacy of blue shark (*Prionace glauca*) cartilage collagen, mention a decrease in wrinkles. A test evaluated the skin’s properties before and after applying a gel with a concentration of 0.125–5.0% added lyophilized hydrolyzed shark cartilage. The same results were obtained in the previously discussed study [42].

Skin surface roughness is an important indicator of healthy, well-moisturized skin with a soft, isotropic texture—an objective of modern dermocosmetics. A visual assessment of the skin condition is only possible in the primary lines. Changes in skin topography are influenced by many internal and external factors, including anatomical location, regional differences, the influence of environmental factors or UV radiation, and changes caused by the body’s own aging process. With age, the primary lines of skin relief deepen, and the number of secondary lines decreases, increasing anisotropy [52]. The results presented here demonstrate the effect of the gel matrix with 0.5 and 1.5% fish CH on the reduction of wrinkle depth compared to placebo as well as R1–R5 roughness values at time T0 compared to values at time T8 in the range of 3.0–26.2%. The reducing effect of a gel matrix with the addition of hydrolysate from chicken gizzards on the texture of the skin around the eyes is also described in [42], where the reduction in roughness parameters was in the range of 34–43%. UV radiation is a major environmental factor that significantly impacts skin conditions, primarily through photoaging mechanisms involving complex neuroimmune and neuroendocrine pathways [53]. The skin’s response to environmental stressors is mediated by the production of various signaling molecules, which play a crucial role in maintaining skin structure and homeostasis [54]. While the beneficial effects of collagen hydrolysates on skin texture improvement have been well-documented in this study, it is possible that in addition to directly affecting skin texture, these substances may indirectly modulate the neuroendocrine balance and thus contribute to protection against environmental stressors. While UV radiation is a major environmental factor in photoaging, collagen hydrolysates may not only enhance collagen synthesis to improve skin texture. Still, they could also potentially support the skin’s natural defenses against UV-induced damage. This could be through the modulation of the skin’s neuroendocrine pathways, which play a critical role in responding to and protecting against environmental stressors. A variety of other anti-aging studies have been conducted with topical formulations containing active ingredients, such as 5% arginine or peptides, with different application times in the periorbital area, and these have shown positive changes in the reduction of wrinkle relief [55,56,57]. Similarly interesting results were obtained with oral supplementation with collagen peptides [58] or cocoa flavonols [59].

Given the promising results, further studies are needed to verify the benefits of topical application of natural ingredients and their combinations, particularly in clinical settings involving both intact and compromised skin barriers in the periorbital area.

## 4. Materials and Methods

### 4.1. Preparation of Collagen Hydrolysates

The following raw materials were used for the preparation of collagen hydrolysates: chicken gizzards (Raciola Ltd., Uherský Brod, Czech Republic), deer tendons (Venison CZ Ltd., Míškovice, Czech Republic), and fish skeleton (Tovačov Fisheries, Tovačov, Czech Republic). Collagen hydrolysates were subsequently prepared from the above raw materials using the below procedure [42]. The preparation of collagen hydrolysate consisted of two steps: (a) preparation of pure collagen; (b) extraction of hydrolysate. For hydrolysate preparation from fish skeletons, pure collagen extraction was supplemented by a demineralization procedure [42]. Chicken gizzards and deer tendons were ground and homogenized into 3 mm particles (SPAR Mixer SP–100 AD–B (Gastrotip, Hradec Králové, Czech Republic), then washed thoroughly in water. The collagen hydrolysate was prepared according to optimized conditions previously published [60]. The organic matter (chicken gizzards and deer tendons) were treated in 0.2 M NaCl (Verkon, Praha, Czech Republic) in a ratio of 1:6 and 0.03 M NaOH (Verkon, Praha, Czech Republic) in a ratio of 1:6 for 2 h and 24 h, respectively, and then dried at 36.0 ± 0.2 °C for 34 to 36 h (VENTICELL 200I, München, Germany) They were further thoroughly degreased with petroleum ether (Verkon, Praha, Czech Republic) and ethanol (Penta, Praha, Czech Republic) (1:1) in a ratio of 1:9 (100 g gizzards or tendons and 900 mL solvent mixture) for 48 h.

The gelatin preparation from fish skeletons was performed according to optimized conditions published by Gál et al. [5]. Separation of inorganic matter in fish skeletons was performed by mixing raw material (gelatin from fish skeletons) in a ratio of 1:10 with HCl (concentration of HCl was 1 wt%) and demineralized with gentle shaking on HS 501 (IKA, Staufen, Germany) at temperature 22.0 ± 1 °C for 48 h; after 24 h, the acid was replaced with new acid. After filtration, the demineralized collagen was washed thoroughly with cold water and dried in VENTICELL 200I (MMM Medcenter, München, Germany) for 24 h at 35 ± 1 °C.

Collagen hydrolysates were extracted from the dried and defatted tissues (chicken, deer, or fish skeletons) by treatment of the raw material with Protamex^®^ (Novozymes, Copenhagen, Denmark). In the first phase, the tissue was mixed with water in a ratio of 1:10 (100 g gizzards or deer tendons and 1000 mL water), and after 20 min, the pH was adjusted to 6.3 ± 0.2 by adding 0.2 M HCl and using a solution of 0.03 or 0.06 NaOH (Verkon, Praha, Czech Republic). Subsequently, as the pH stabilized, 0.6% of Protamex^®^ enzyme was added, and the mixture was shaken intensively for 30 h at room temperature (HS 501, IKA; Staufen, Germany). In the second phase, the mixture was mixed with water in a ratio of 1:8 (100 g of gizzards, tendons or skeletons, and 800 mL of distilled water), and the tissue was heated to 66 °C (dt/dτ = 10 °C/min) and extracted at this temperature for 2 h. In the last step, the mixture was filtered several times through PA fabric, and the resulting hydrolysate solution was dried in a thin film at 45.0 ± 0.2 °C for 24 to 36 h (VENTICELL 200I, München, Germany). All dried hydrolysate films were ground in a mortar (ETA 0010, Praha, Czech Republic) and homogenized to a very fine powder of size particles 0.1–0.2 mm.

### 4.2. Characterisation of Collagen Hydrolysates

#### 4.2.1. Determination of Dry Matter Content and Ash Content

The gravimetric method determined the dry matter content by weighing approximately 1.3 g of CH (chicken gizzard hydrolysate, deer tendon hydrolysate, and fish skeletal hydrolysate) into two dishes twice. The samples were placed in a ULP 400 oven (Memmert, Schwabach, Germany) at 103 ± 1 °C for 12 h. They were then placed in a desiccator to cool and weighed on an ALS 250-4A balance (Kern, Balingen, Germany). The dry matter content was calculated according to the Equation (1):

(1) $$S=\frac{m}{m_{0}}\cdot 100$$

where:

m—weight of the sample after drying [g];

m_0_—weight of the sample before drying [g].

The ash content was determined by weighing approximately 1.3 g of each CH sample (hydrolysate from chicken gizzard, deer tendon, and fish skeleton) in duplicate in annealing crucibles. The samples were first burned over a charcoal burner and then placed in an L9/11 muffle furnace (Thermo Fisher Scientific, Waltham, MA, USA) for 4 h and annealed at 650 ± 1 °C. After cooling, the crucibles were weighed, and the ash content was calculated as follows (1):

(2) $$P=\frac{m}{m_{0}}\cdot 100$$

where:

m—weight of the sample after annealing [g];

m_0_—weight of the sample before annealing [g].

#### 4.2.2. Determination of Clarity and pH

The purity and pH of CH were determined for its 6.67% solution in demineralized water [61]. The prepared solutions were placed in cuvettes in a Helios spectrophotometer (Thermo Fisher Scientific, Waltham, MA, USA), and their transmittance at 640 nm—i.e., the amount of light passing through the sample—was measured. The purity of the CH solutions was also determined after purification. For this purpose, the individual CHs were boiled in distilled water and then dried. A pH meter PH60S-Z (Apera Instruments, Columbus, OH, USA) was used to measure the pH of the (6.67%) CH solutions. The clarity of a 6.67% gelatin solution was determined at 45 °C by measuring the percent transmittance through a 1 cm cuvette at 640 nm. Calibration with distilled water was performed before measurement.

#### 4.2.3. Determination of Antioxidant Activity

Antioxidant activity (AA) was measured by the DPPH method, which uses 2,2-diphenyl-1-picrylhydrazyl (MedChem Express, Monmouth Junction, NJ, USA) as an antioxidant reagent. A concentration series of five solutions of each CH species (chicken gizzard hydrolysate, deer tendon hydrolysate, and fish skeletal hydrolysate) in demineralized water in the 2–10 mg∙mL^−1^ was prepared. In addition, one liter of a 0.2 mM solution of DPPH in 96% ethanol was prepared. The absorbance of the DPPH solution was then measured at 517 nm using a Helios spectrophotometer (Thermo Fisher Scientific, Waltham, MA, USA). For the determination of AA alone, it was necessary to prepare CH solutions from each concentration series by adding 96% ethanol and 0.2 mM DPPH solution (1 mL of CH solution at 2, 4, 6, 8, or 10 mg∙mL^−1^ and 1 mL of 96% ethanol and 0.25 mL of 0.2 mM DPPH solution). The prepared solutions were then kept in the dark for 30 min. Their absorbance was then measured at a wavelength of 517 nm. From the resulting absorbance values, the antioxidant activity was calculated as follows (3):

(3) $$AA=\frac{A_{DPPH}-A_{vz}}{A_{DPPH}}\cdot 100$$

where:

A_DPPH_—absorbance of 0.2 mM DPPH solution in ethanol;

A_vz_—absorbance of the sample in ethanol and DPPH solution after 30 min.

#### 4.2.4. FTIR Analysis

The functional groups of hydrolysates were determined by Fourier transform infrared (FTIR) spectroscopy using the Bruker ALPHA instrument (Bruker GmbH, Vienna, Austria), as described by Prokopová et al. [26]. The measurements were performed using the ATR method, with the platinum crystal always oriented on the side facing the lamp during photo exposure. Only the background without a gelatin sample was used as a control. Spectra ranging from 400 to 4000 cm^−1^ were collected for 32 running scans.

#### 4.2.5. Molecular Weight Distribution

The molecular weight distribution of gelatin samples was determined by gel permeation chromatography with refractometric detection (GPC-RID). The analytical method consisted of weighing a 2.00 ± 0.01 mg powder sample and dissolving it in 1 mL of 0.1 mol/l phosphate buffer in a sealed vial at 20.0 ± 2.0 °C for 4 h. A total of 100 μL of the sample was injected into the analytical apparatus of a Waters HPLC Breeze 2414 differential refractometer (Waters, Milford, MA, USA) with OHpak SB-806M HQ column (300 × 8 mm, 13 µm) + OHpak SB-804 HQ. The measurement process was carried out at 40.0 ± 1.0 °C and the flow rate of the solution was 1 mL/min. The system was calibrated using pullulan standards in the 667–344,000 Da range.

### 4.3. Surface Tension and Contact Angle Determination

The surface tension of the samples (demineralized water and 0.5 and 1.5% CH solutions in water) was measured on an EasyDyne K20 tensiometer (Krüss, Hamburg, Germany) according to the Wilhelmy method. Each sample was measured three times.

The contact angle measurements were carried out with a Drop Angle Meter DM 300 (Courage and Khazaka Electronics, Cologne, Germany), with which cosmetic formulations can be measured directly on the test person’s skin. The samples for the contact angle measurements of 40 µL consisted of solutions (0.5 and 1.5%) of individual collagen hydrolysates (hydrolysate from chicken gizzards, deer tendons, and fish skeletons) in water. The measurements were carried out in triplicate for each sample.

### 4.4. Preparation of Formulations with Collagen Hydrolysate

The emulsion matrix was made by weighing the raw materials from the aqueous and oil phase (Table 8) into a beaker, which was then heated in a water bath to approximately 60 °C. The aqueous phase was stirred into the oil phase using a Heidolph RZR 2020 homogenizer (IKA, Staufen, Germany) at 2000 rpm for about 10 min, i.e., until the entire mixture was cooled. The pH of the prepared emulsion was always about 5.5–6.0 ± 0.1 (5 wt% aqueous NaOH; Penta, Praha, Czech Republic). A reference emulsion without any collagen hydrolysate was prepared using the same procedure.

The gel formulations (Table 8) were composed of the carbomer (Míča a Harašta, Blansko, Czech Republic), aqua, sodium hydroxide (Penta, Praha, Czech Republic), and any collagen hydrolysate (TBU in Zlín, Zlín, Czech Republic). They were prepared according to the following description: each active ingredient was dissolved in a specific amount of water, and then the carbomer (0.6 wt%), methylparaben (0.2 wt%), and propylparaben (0.1 wt%) were added. The formulation was left at rest for 24 h at room temperature to swell the carbomer thoroughly. Subsequently, the gel was homogenized by stirring it on a laboratory stirrer (Heidolph, Schwabach, Germany), first for 30 min at 160 rpm and then for 10 min at 360 rpm at the close. The gel was neutralized by 10wt% aqueous NaOH during the mixing process with a pH value of 6.0 ± 0.1. A reference gel without any collagen hydrolysate was prepared using the same procedure.

### 4.5. pH Measurement

A pH meter (pH Spear Waterproof, Waltham, MA, USA) with a measurement accuracy of ±0.1 pH was used to measure the pH of the produced emulsions and gels. All measurements were taken at least three times for each value.

### 4.6. Microbiological Analyses

First, the microbial contamination was determined by total viable counts (TVC) and yeasts and mould counts (YMC). The sample (0.1 g) was aseptically mixed with 0.9 mL of sterile saline solution and shaken for 4 min on vortex (3000 rpm/min). Decimal dilutions were poured by or plated on Plate Count Agar (PCA) for TVC determination or Chloramphenicol Yeast Glucose Agar (CYGA) for YMC determination; both media were supplied by Himedia Laboratories Pvt. Ltd., Mumbai, India. PCA plates were incubated for 48 h at 30 ± 1 °C, and CYGA plates were placed at 22 ± 1 °C for 5 days.

Second, a preservative efficacy test was performed with *Pseudomonas aeruginosa* ATCC 27853 according to ISO 11930:2019 [22]. The bacterial strain was obtained from the Czech Collection of Microorganisms (Brno, Czech Republic). Samples (5 g) were inoculated by 50 µL bacterial suspension (1.5∙108 CFU·mL^−1^), mixed well, and stored at 22 ± 1 °C. Immediately (0 days) and after 7, 14, and 28 days of storage, the microbiological analysis as described earlier was determined with Nutrient Agar (Himedia Laboratories Pvt. Ltd., Mumbai, India). The plates were incubated at 37 ± 1 °C for 24 h. All experiments were performed in triplicate.

### 4.7. Skin Diagnostics

The effect of the prepared emulsion matrices on the skin of the inner forearm’s volar side and the face’s periorbital location was monitored using non-invasive bioengineering instrumental methods quantifying the biophysical parameters of the skin and dermal micro relief.

#### 4.7.1. Study Design of Effectiveness of CH Emulsion and Gel Matrices on Volar Forearm Skin

Eight volunteers aged 40 ± 2 without health problems participated in the skin diagnosis. The volunteers were familiar with the goal of the experiment and its progress. Nevertheless, the selection of volunteers and the testing procedure were under international ethical principles of biomedical research with human participants [62].

The study was conducted in a one-sided, blinded, placebo-controlled design with a comparison of untreated skin and skin treated with SDS solution. Measurement of bio-physical parameters (hydration, TEWL, and pH) of the skin took place in an air-conditioned room (temperature 22–24 °C, relative humidity 45–50%) after twenty minutes of volunteer acclimatization. Both volar sides of the forearm were divided into 10 test sites, each with an area of 8 cm^2^. The first place of the volar side of the forearm remained untreated, which served as the so-called control for visual comparison because of possible skin irritation. The second place remained also without the application of emulsion formulations. These two areas served for the initial measurement (t = 0 h). All other sites were pre-treated with a 0.5 wt% solution of sodium dodecyl sulfate (SDS, Sigma-Aldrich, St. Louis, MO, USA) in saline and left for four hours. After this, 0.1 mL of each of eight emulsion and gel formulations (E, EDH, ECHH, EFH, G, GDH, GCHH, and GFH) were applied to one of eight pre-treated sites. The effect of these formulations was monitored after 1, 2, 3, 4, 24, and 48 h.

#### 4.7.2. Study Design of Effectiveness of Fish CH Gel Matrices on Periorbital Region

For this part of the study, a gel matrix with collagen fish hydrolysate of 0%, 0.5%, and 1.5% were selected. Fifteen women aged 45 ± 3 years participated in the study, divided into three groups applying the above gel formulations in the periorbital area. The study was conducted under the same laboratory and ethical conditions as the part performed on the inner side of the volar forearm but timed in intervals of eight weeks, designated as T0–T8. The volunteers applied the respective formulations to a defined part of the periorbital area treated with micellar water (Laboratoires dermatologiques d’URIAGE, France) twice daily in the morning and evening when they were instructed not to treat this area with any other cosmetic products or other techniques. The skin condition of the right and left periorbital areas of the face was quantified by monitoring hydration (measured 5 times), TEWL (measured 15 times; the first five values were neglected due to adaptation to fluctuating temperature and humidity values between the surface of the *stratum corneum* and the inner space of the probe chamber), pH (measured 3 times), and scanning the number of wrinkles (1 scan). In a given area, the skin relief with the number of wrinkles was scanned by the volunteers using silicone replicas and a camera.

#### 4.7.3. Non-Invasive Instrumental Bioengineering Methods and Skin Surface Characteristics

The MPA station (Courage and Khazaka Electronic GmbH, Cologne, Germany) was the platform for diagnosing the effect of prepared emulsion matrices on the skin. To measure the water content in the *stratum corneum*, the Corneometer^®^ CM 825 corneometric probe (Courage and Khazaka Electronic GmbH, Cologne, Germany) was used. It is based on evaluating changes in electrical capacitance corresponding to the water content in the skin’s *stratum corneum*. Hydration was measured five times at each marked site of the volar side of the forearm, and the mean value with standard deviation was expressed in corneometric units (c. u.) according to the scale. A < 30 c. u. scale corresponds to extremely dry skin, 30–40 c. u. to dry skin, and normally hydrated skin to values of >40 c. u. [45].

Another tested parameter was TEWL, which was monitored by the Tewameter^®^ TM 300 probe (Courage and Khazaka Electronic GmbH, Cologne, Germany) at each test site 15 times; the first 5 values were neglected due to the equalization of temperature and humidity in the probe chamber and the skin surface of the volunteers’ volar forearm. From the last 10 values, the mean and standard deviation were calculated. In principle, the method determines the flow of water vapor above the *stratum corneum* into the space of a cylindrical chamber with two pairs of sensors for temperature and relative humidity. TEWL is calculated from the difference between the two measurement points using Fick’s law of diffusion and displayed in grams per hour per square meter. The interpretation of the results was based on a scale that characterizes the condition of the skin in the range of 0–10 g·m^−2^·h^−1^ for very healthy conditions, 10–15 g·m^−2^·h^−1^ for healthy conditions, 15–25 g·m^−2^·h^−1^ for normal condition, 25–30 g·m^−2^·h^−1^ for strained skin and above 30 g·m^−2^·h^−1^ for skin in critical condition [63].

To determine the acidity of the skin surface, the skin-pH-meter^®^ PH 905 was used (Courage and Khazaka Electronic GmbH, Cologne, Germany). The specially designed probe comprises a flat-topped glass electrode for full skin contact connected to a voltmeter. The system measures potential changes due to the activity of hydrogen cations surrounding the very thin layer of semisolid forms at the top of the probe. The pH was measured three times at each marked site of the volar side of the forearm, and the mean value with standard deviation was. The changes in voltage are displayed as pH, which has been interpreted as acidic for the range 3.5–4.3; normal 4.5–5.5; high > 5.7 [48].

A topographic method of profile imaging using silicone replicas was used to monitor changes in skin microrelief in the periorbital region. An adhesive mold (Courage and Khazaka Electronic GmbH, Cologne, Germany) was glued to the measured periorbital area on both sides of the face, defining the site for the application of a silicone compound prepared by mixing silicone base and catalyst (Courage and Khazaka Electronic GmbH, Cologne, Germany) in a plastic container in a 1:1 ratio while sucking out the air present using a Visiometer^®^ Vacuum Pump VP45 (Courage and Khazaka Electronic GmbH, Cologne, Germany). The applied two-component silicone compound was immediately covered with a cover sheet and cured for 10 min. The fabricated replica was glued onto a paper template with identification data. A calibrated Skin-Visiometer^®^ SV 700 (Courage and Khazaka Electronic GmbH, Cologne, Germany), which works by measuring the intensity of light transmitted through the silicone replica using the Lambert–Beer law, was inserted into the calibrated device. The corresponding program was used to evaluate the roughness parameters R1–R5 on the circular area of the replica surface, which compensated for the influence of the wrinkle direction. The parameter R1 indicates the distance between the highest and lowest point of the scanned area, R2 indicates the maximum roughness calculated from different values of the roughness segments, R3 represents the average roughness parameter of the five highest and five lowest segments, R4 expresses the maximum height of the profile related to the length of the evaluated area, R5 presents the difference of the height of the actual profile from the average profile [64]. The detailed procedure of the method is presented in the publication by Prokopová et al. [42].

Images of the skin were also taken with a unique Visioscope^®^ PC 35 camera, evaluated with the Skin Competence System software (version 1.0, Courage and Khazaka Electronic GmbH, Cologne, Germany), which allowed the percentage quantification of wrinkles in the periorbital region.

### 4.8. Sensory Analysis

The organoleptic properties of the prepared emulsion and gel formulations were assessed by a panel of 11 assessors trained according to ISO 8586:2023 [65], who were acquainted with the course and goal of the sensory evaluation. The evaluation conditions were ensured according to the requirements specified in ISO 6658:2017 [66] and ISO 8589:2007 [67]. The samples were presented randomly at a controlled temperature of 22 ± 1 °C under normal lighting conditions in a sensory laboratory equipped with sensory cubicles.

The samples for sensory evaluation were coded as follows: A—sample without the addition of collagen hydrolysate; B—sample with the addition of 0.5% chicken collagen hydrolysate; C—sample with the addition of 0.5% deer collagen hydrolysate; D—sample with the addition of 0.5% fish collagen hydrolysate; E—sample with the addition of 1.5% of chicken collagen hydrolysate; F—sample with the addition of 1.5% deer collagen hydrolysate; and G—sample with the addition of 1.5% fish collagen hydrolysate.

The sensory assessment questionnaire included a ranking test [68]. The ranking test investigated the spreadability of seven gel and emulsion formulations (1—the best spreadable sample; 7—the least spreadable sample), as well as absorbency (1—excellent and rapid absorption; 7—not absorbed, leaves a strongly sticky film), smell (1—the best smell; 7—the least smell sample), and color (1—excellent; 7—unsatisfactory). Further, the preference for gels or emulsions was assessed (1—the most preferred sample; 7—the least preferred sample).

### 4.9. Statistical Analysis and Data Processing

Microbiological results and diagnostically gained biophysical characteristics were statistically analyzed using Microsoft Office Excel (version 10, Microsoft, Santa Rosa, California, USA) and presented as an arithmetic mean and standard deviation. Subsequently, the values were tested by paired *t*-test for statistical significance (*p* < 0.05) compared to pre-treatment values in individual time intervals for the study performed on the volar forearm. The efficacy of the matrices without the CH addition/placebo and with the addition of 0.5% a 1.5% fish CH in the periorbital region was determined by relative changes of biophysical parameters by the differences of the means: T8–T0.

The Friedman test evaluated the ranking tests of sensory analyses and the existence of differences between samples was determined using Neményi’s method. The paired comparison test was assessed using the Fisher test criterion. The results of the sensory analysis were processed at a 1% significance threshold (*p* < 0.01) by Unistat 5.5 software (Unistat Ltd., London, UK).

## 5. Conclusions

Research into the structural and biophysical properties of chicken, deer, and fish hydrolysates has shown that they are suitable as alternative biomaterials to conventional bovine collagen for human and veterinary cosmetics development. The differences observed between the various collagen sources may be due to the different types of original by-products and the conditions under which the collagen hydrolysates are produced (different extraction and purification methods and conditions). Collagen hydrolysates from alternative raw material sources form solutions that wet the skin surface well and are suitable for producing cosmetic matrices for emulsion and gels. The high antioxidant activity of collagen hydrolysates and the moisturizing effect of emulsion matrices contribute to improved skin condition. Cosmetic formulations containing fish collagen hydrolysate have been shown to have a positive effect on reducing anisotropy. The higher degree of polydispersity of collagen hydrolysates proved beneficial for cosmetic applications as an improved and restored skin barrier was observed after applying the tested formulations. Topical procedures may be an appropriate complementary or alternative approach to aesthetic modalities leading to periorbital rejuvenation.

## 6. Limitations

This study had several limitations that may affect the generalizability of its findings. The first limitation was the relatively small sample size, which may limit the ability to validate the efficacy of the formulations prepared with collagen hydrolysates on the volar side of the forearm using objective methods measuring biophysical parameters such as hydration, TEWL, and pH. Another part of the study, conducted in the periorbital region, also monitored the same biophysical parameters and changes in skin microrelief. However, neither part of this study evaluated parameters that could provide further insight into the cellular or molecular mechanisms involved in all layers of the *epidermis* that may underlie the observed changes.

## Figures and Tables

**Table 1:** Determination of dry matter and ash content of prepared CH solutions.

Collagen Hydrolysate	Dry Matter [% *w*/*w*]	Ash Content [% *w*/*w*]
Chicken	92.36 ± 0.12	8.10 ± 0.05
Deer	92.9 ± 0.09	0.66 ± 0.06
Fish	95.87 ± 0.02	24.89 ± 0.09

**Table 2:** Determination of the clarity and pH of CH solutions.

Collagen Hydrolysate	Clarity [% *w*/*w*]		pH
	Before Purification	After Purification	
Chicken	2.8	3.6	6.72
Deer	5.3	6.8	7.08
Fish	0.9	1.9	5.21

**Table 3:** Antioxidant activity of prepared CH solutions.

Collagen Hydrolysate	Chicken	Deer	Fish
Concentration of CH [mg·mL^−1^]	Antioxidant Activity [%]		
2	76.83	65.77	85.24
4	54.56	58.57	81.64
6	49.19	35.45	80.17
8	32.71	29.36	73.53
10	26.42	26.12	72.06

**Table 4:** Molecular weight characteristics for collagen hydrolysate, including PDI.

Collagen Hydrolysate	M_w_^1^ [g·mol^−1^]	M_n_^2^ [g·mol^−1^]	PDI [−]
Chicken	95.400	5900	16.2
Deer	169.000	28.500	5.9
Fish	7500	1000	7.9

^1^ Weight—average molecular weight. ^2^ Number—average molecular weight.

**Table 5:** Surface tension and contact angles of CH solutions.

Collagen Hydrolysate	Concentration of CH [%]		Contact Angle [°]	
		Surface Tension [mN∙m^−1^]	θ [°]	θ1 [°]
Chicken	0.5	48.8 ± 1.9	38.1 ± 0.1	19.1 ± 0.1
	1.5	47.1 ± 0.7	52.1 ± 0.1	26.0 ± 0.1
Deer	0.5	41.3 ± 0.2	55.2 ± 0.1	27.6 ± 0.1
	1.5	45.2 ± 1.3	53.7 ± 0.1	26.9 ± 0.1
Fish	0.5	40.1 ± 1.0	63.0 ± 0.1	31.5 ± 0.1
	1.5	37.3 ± 0.5	44.0 ± 0.1	22.0 ± 0.1

**Table 6:** Preservative efficacy test of CH emulsions with *Pseudomonas aeruginosa* (CFU·g^−1^).

	Concentration of CH [%]	Days			
		0	7	14	28
Chicken	0.5	5.5 × 10^5^	6.2 × 10^4^	3.3 × 10^3^	<10
	1.5	6.3 ×∙10^5^	8.1 × 10^4^	1.3 × 10^4^	<10
Deer	0.5	4.1 ×∙10^5^	2.7 × 10^4^	1.2 × 10^3^	<10
	1.5	6.3 ×∙10^5^	7.6 × 10^4^	1.1 × 10^3^	<10
Fish	0.5	1.1 ×∙10^5^	1.4 × 10^4^	6.1 × 10^3^	<10
	1.5	8.9 ×∙10^5^	9.3 × 10^3^	7.0 × 10^2^	<10

**Table 7:** Rank sums for the sensory properties of the ranking test of gels and emulsion samples.

Parameters	Spreadability	Absorbency	Odor	Color	Preference
Formulation	Gel/Emulsion	Gel/Emulsion	Gel/Emulsion	Gel/Emulsion	Gel/Emulsion
A	34/41	36/32	16/47	12/28	23/33
B	39/66	37/66	50/66	23/66	42/66
C	59/22	50/30	27/26	31/33	38/27
D	33/36	4030	36/31	53/25	30/28
E	40/40	38/39	61/29	52/38	48/38
F	72/57	73/60	60/63	61/48	67/61
G	31/72	34/71	62/55	76/67	60/73

A—sample without the addition of collagen hydrolysate; B—sample with the addition of 0.5% chicken collagen hydrolysate; C—sample with the addition of 0.5% deer collagen hydrolysate; D—sample with the addition of 0.5% fish collagen hydrolysate; E—sample with the addition of 1.5% of chicken collagen hydrolysate; F—sample with the addition of 1.5% deer collagen hydrolysate; G—sample with the addition of 1.5% fish collagen hydrolysate.

**Table 8:** Characteristics of model emulsion formulations (E, G, EH, and GH).

	Base Emulsion Matrix (E)			
Aqueous phase	Ingredients (INCI ^a^)	[wt%]	Function	Supplier
	*Aqua*	Ad 100	Solvent	TBU in Zlín, Zlín, Czech Republic
	*Aloe Barbadensis Extract*	2	Regenerating/Revitalizing/Moisturizing	Kosmetické suroviny Ltd., Praha, Czech Republic
	*Glycerin*	4	Humectant	Kosmetické suroviny Ltd., Praha, Czech Republic
	*Methylparaben*	0.2	Preservative	Sigma-Aldrich (St. Louis, MO, USA)
	*Propylparaben*	0.1	Preservative	Sigma-Aldrich (St. Louis, MO, USA)
Oil phase	*Prunus Amygdalus Dulcis Oil*	10	Emollient/Moisturizing/ Skin Conditioning	Libor Baránek, Bojkovice, Czech Republic
	*Butyrospermum Parkii Butter*	4	Skin conditioning	Kosmetické suroviny Ltd., Praha, Czech Republic
	*Cera Alba*	4	Emollient/Emulsifier	Kosmetické suroviny Ltd., Praha, Czech Republic
	*Theobroma Cacao (Cocoa) Seed Butter*	2	Emollient/Protective	Kerfoot Group, Northallerton, United Kingdom
	*Ricinus Communis Seed Oil*	2	Solvent/Emollient	Míča a Harašta, Blansko, Czech Republic
	Olivoil Avenate Emulsifier^®^ (*Aqua*, *Glyceryl Oleate*, *Cetearyl Alcohol*, *Glyceryl Stearate*, *Potassium Olivoyl Hydrolysed Oat Protein*)	12	Emulsifier/Emollient	Kosmetické suroviny Ltd., Praha, Czech Republic
			Gel matrix (G)	
	*Carbomer*	0.6	Gel Forming Agent	Míča a Harašta, Blansko, Czech Republic
	*Methylparaben*	0.2	Preservative	Sigma-Aldrich (St. Louis, MO, USA)
	*Propylparaben*	0.1	Preservative	Sigma-Aldrich (St. Louis, MO, USA)
	*Aqua*	Ad 100	Solvent	TBU in Zlín, Zlín, Czech Republic
	*Sodium Hydroxide*	qs	Adjustment of pH/ Viscosity	Penta, Praha, Czech Republic
			Addition of Collagen Hydrolysate (H)	
	*Collagen hydrolysate*	0.5/1.5	-	TBU in Zlín, Zlín, Czech Republic

^a^ INCI (International Nomenclature of Cosmetic Ingredients).

## Data Availability

Data are contained within the article.
